# Supplementary material for: Methyl and Ethyl Ethers of Glycerol as Potential Green Low-Melting Technical Fluids
Source: Molecules. 2023 Nov 8;28(22):7483. doi: 10.3390/molecules28227483 (PMC10672826; doi:10.3390/molecules28227483)
Supplement: Supplementary file 1 [file molecules-28-07483-s001.zip › molecules-2698342-supplementary.pdf]

Supporting Information

Manuscript Title: Methyl and ethyl ethers of glycerol as potential green low-melting technical fluids

Manuscript Authors: V.O. Samoilov, V.M. Lavrentev, M.U. Sultanova, D.N. Ramazanov, A.A. Kozhevnikov, G. A. Shandryuk, M.I. Kniazeva, A.L. Maximov

Authors' affiliation: Topchiev Institute of Petrochemical Synthesis, Russian Academy of Sciences, Leninsky pr. 29, Moscow 119991, Russia

Contents:

Figures S1–S4. The chromatograms of the synthetic samples of glycerol ethers.

Figures S5–S11. The DSC curves for pure ethers and aqueous solutions.

Table S1. Density measurements: calibration, raw data and uncertainties.

Table S2. Viscosity measurements: calibration, raw data and uncertainties.

The chromatograms of the synthetic samples of glycerol ethers.

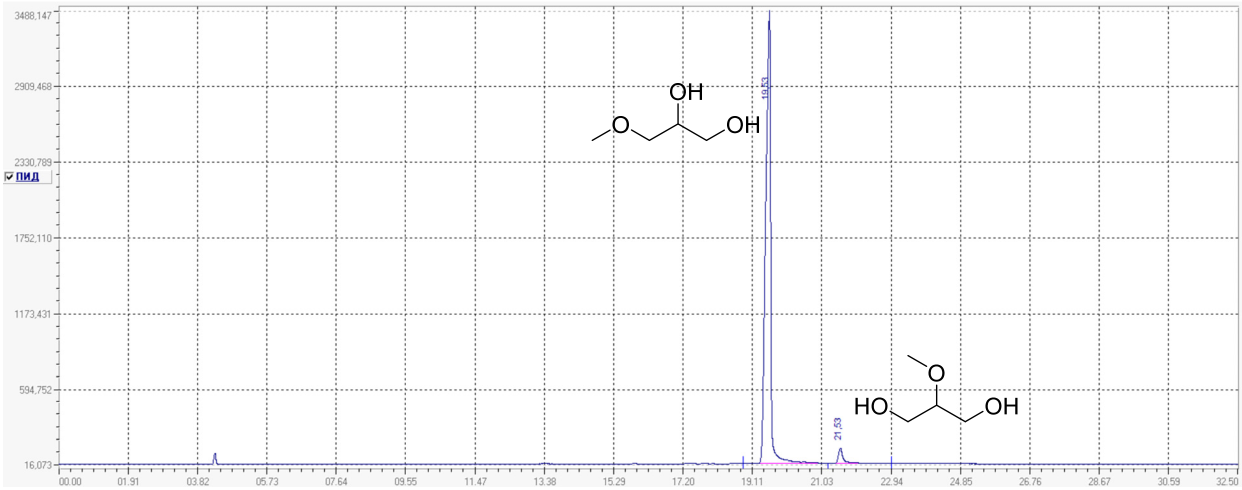

Figure S1. The GC-FID chromatogram for GMME sample.

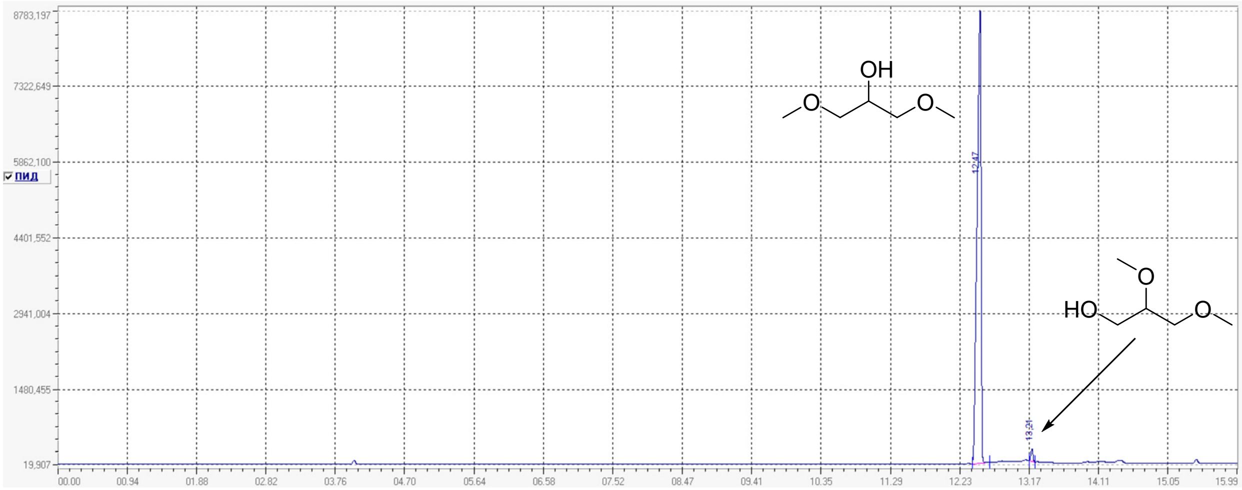

Figure S2. The GC-FID chromatogram for GDME sample.

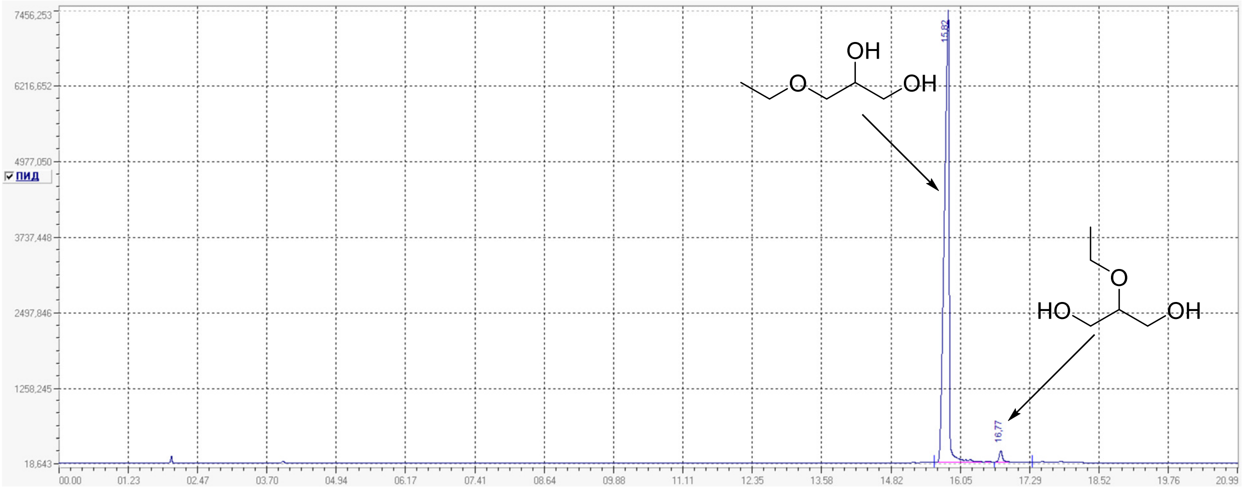

Figure S3. The GC-FID chromatogram for GMEE sample.

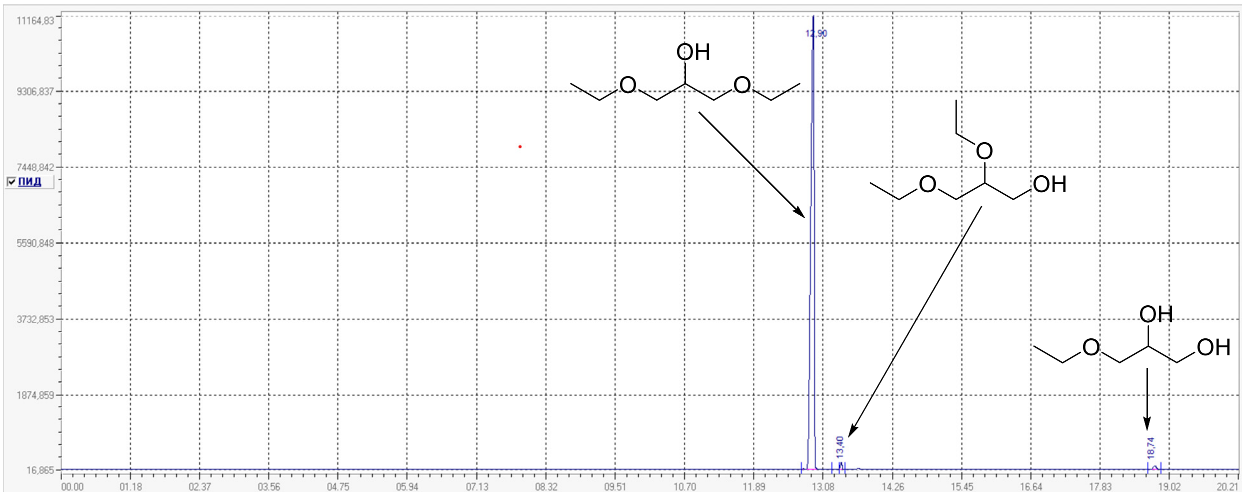

Figure S4. The GC-FID chromatogram for GDEE sample.

The DSC curves for pure ethers and aqueous solutions.

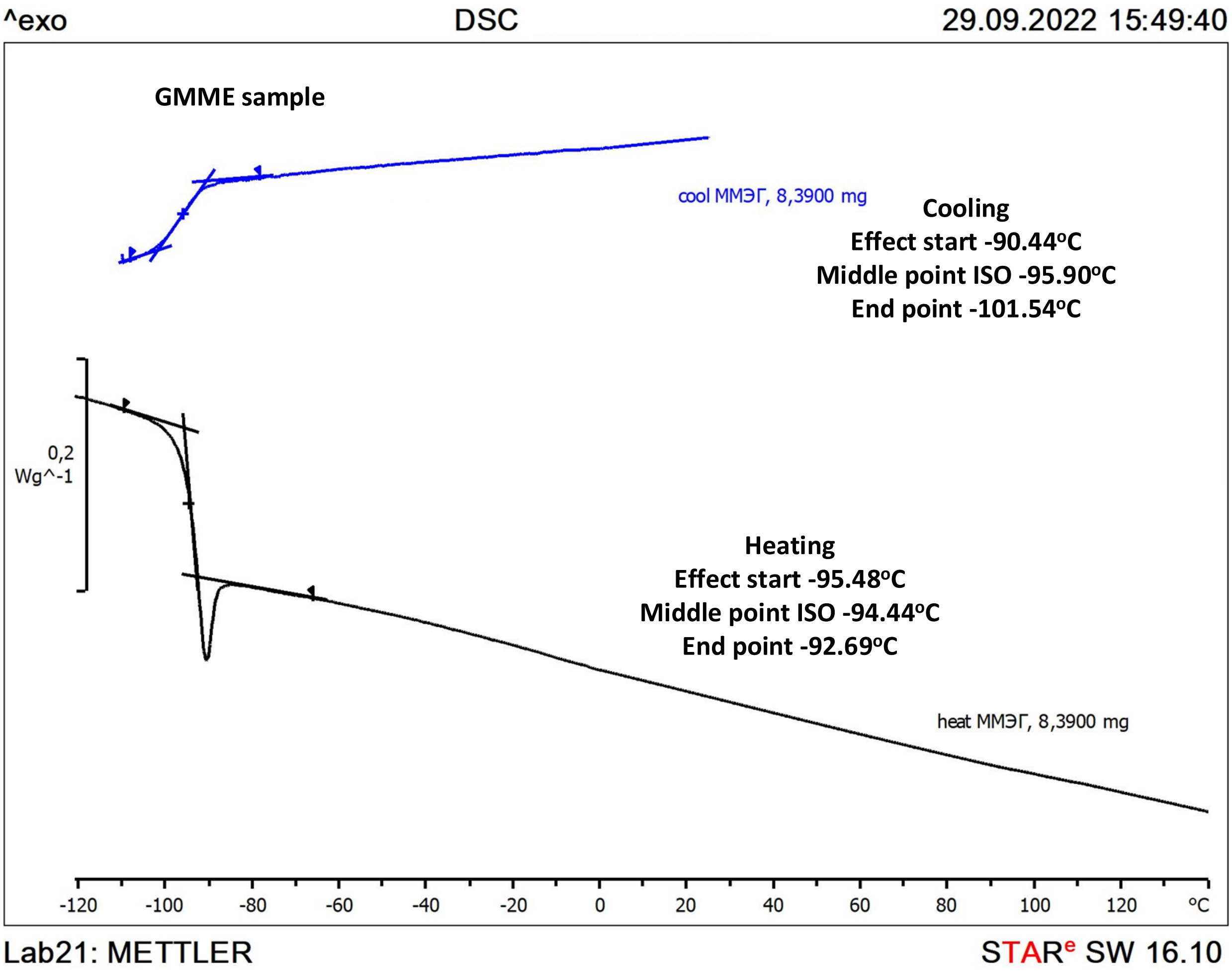

Figure S5. The DSC curves for GMME sample (low-temperature phase transition screening).

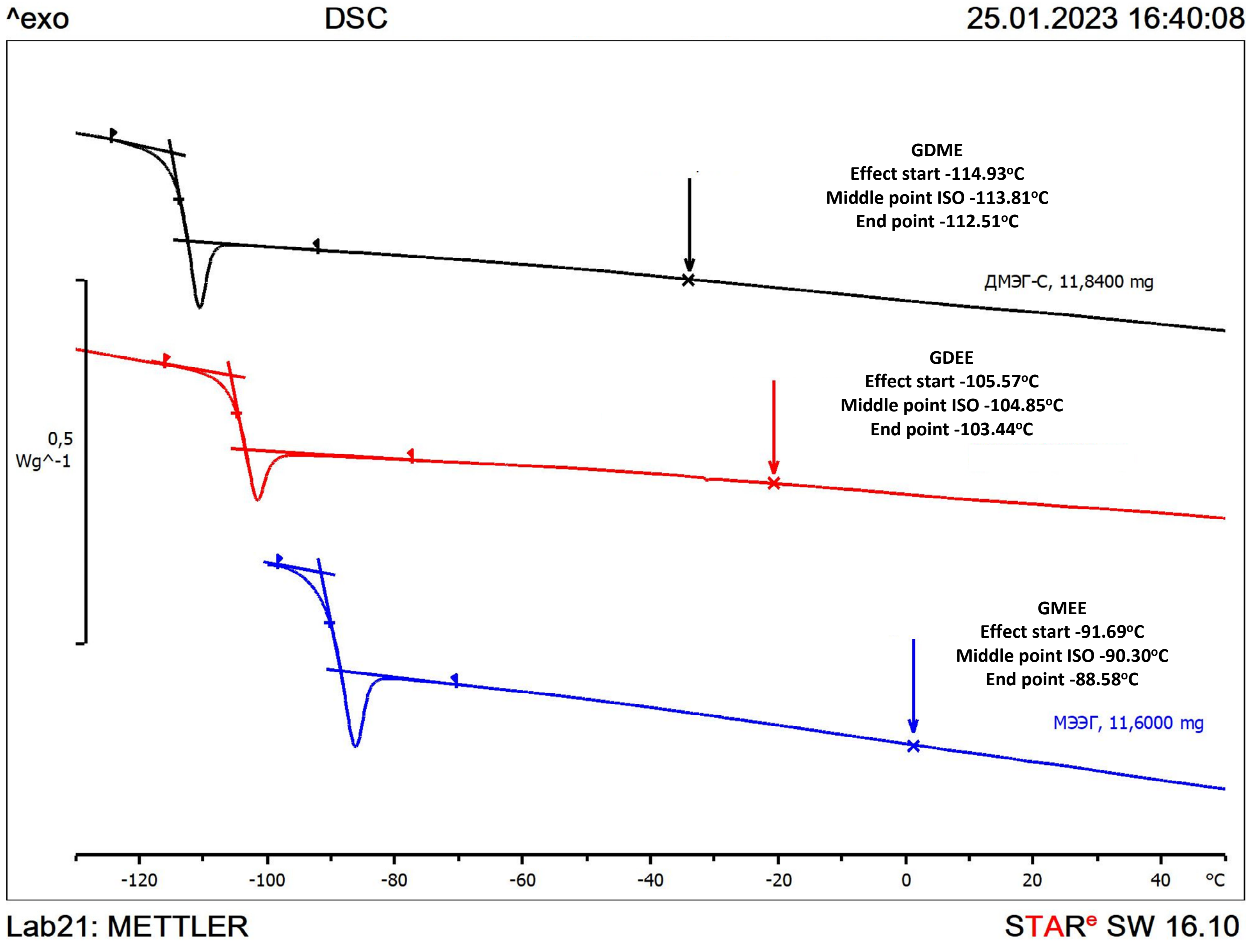

Figure S6. The DSC curves for GDME, GDEE and GMEE samples (low-temperature phase transition screening).

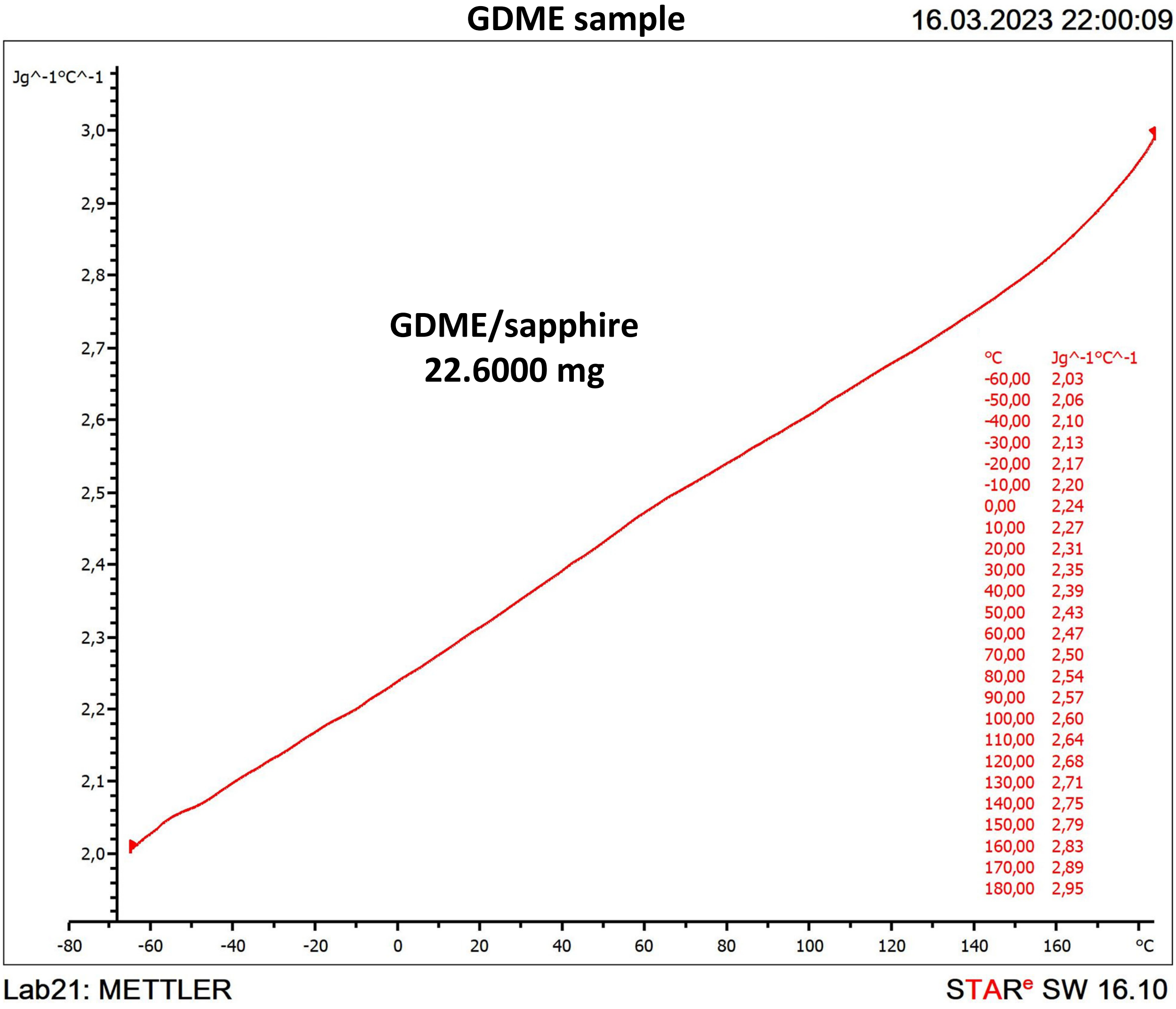

Figure S7. The DSC curve for GDME sample (specific heat capacity measurement).

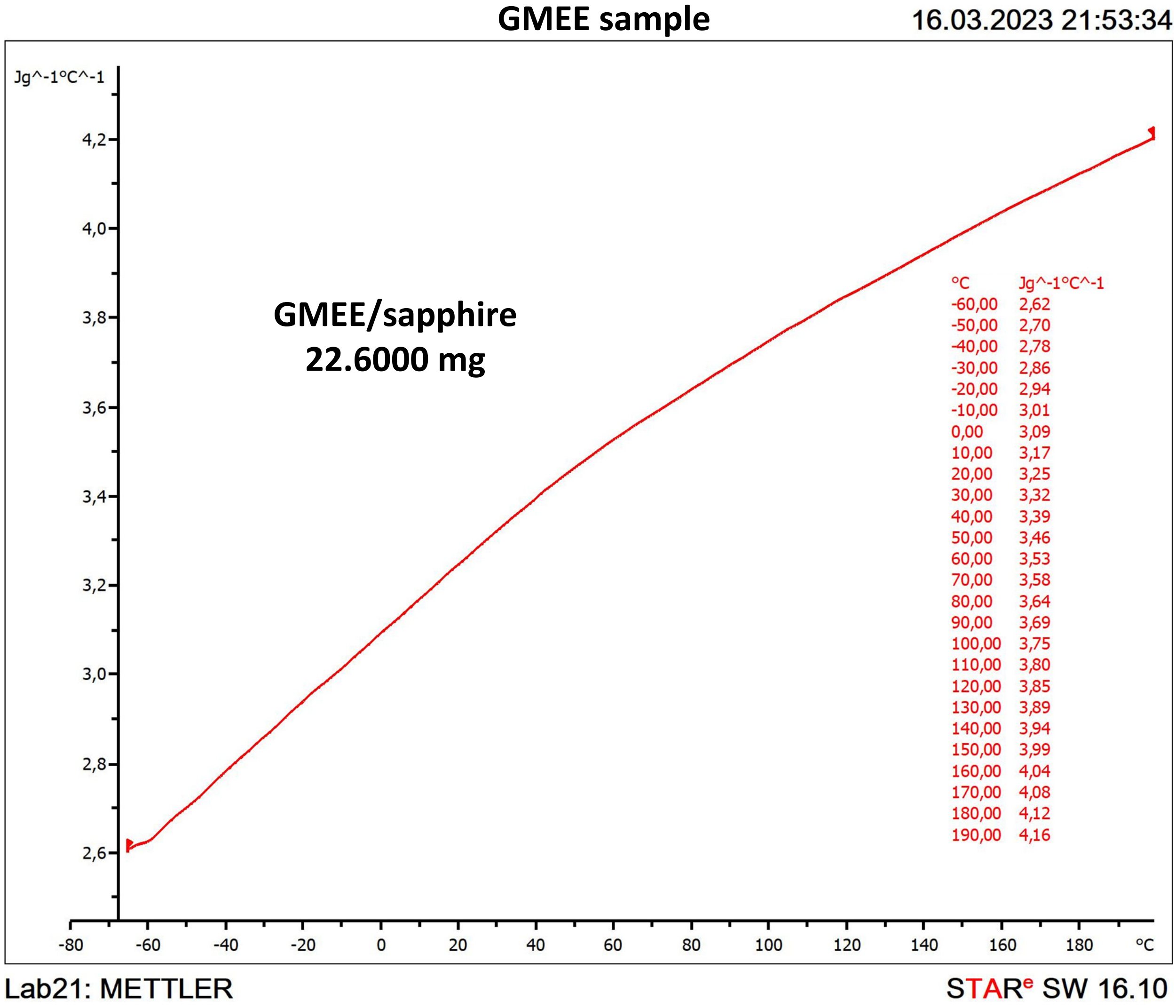

Figure S8. The DSC curve for GMEE sample (specific heat capacity measurement).

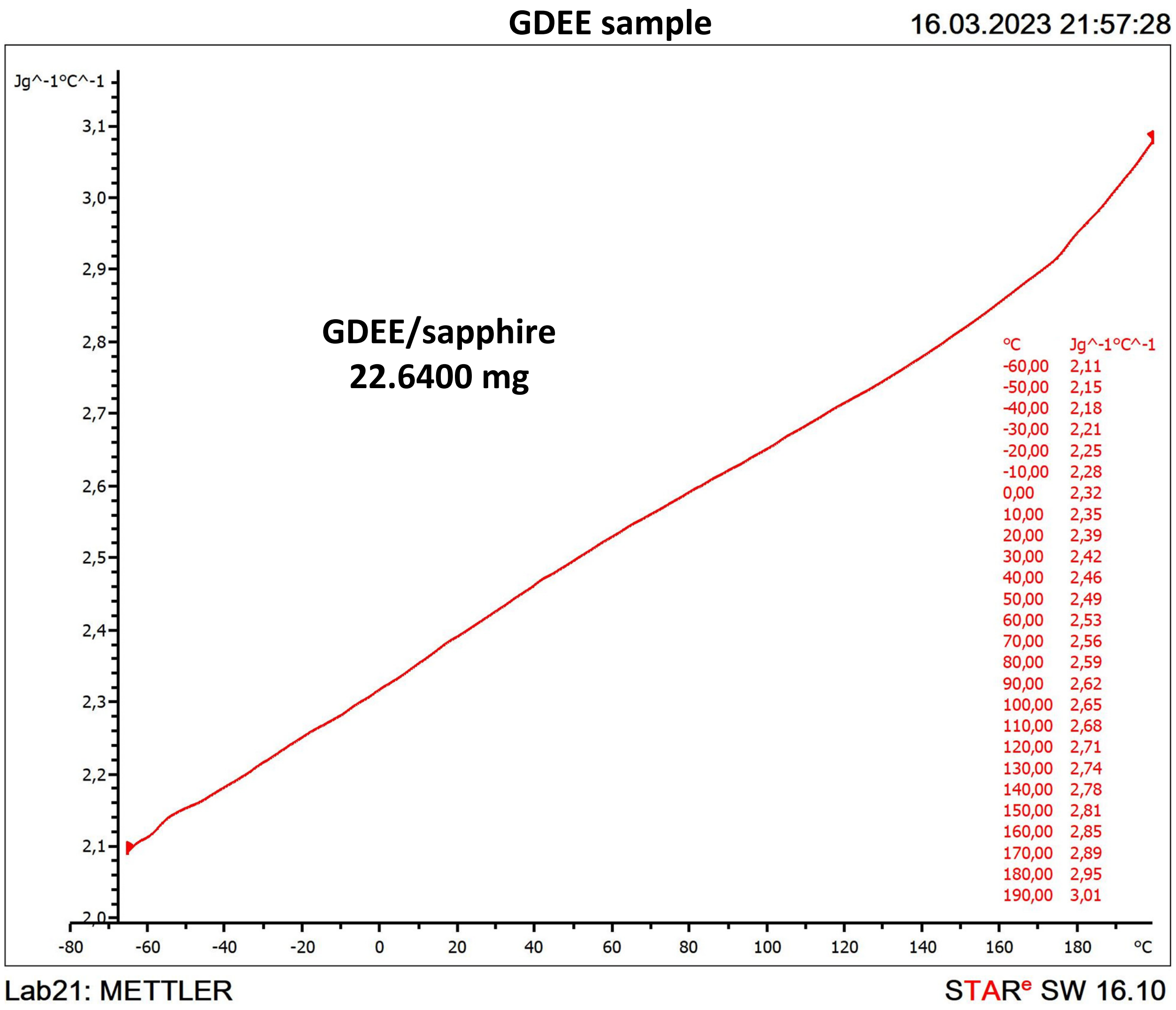

Figure S9. The DSC curve for GDEE sample (specific heat capacity measurement).

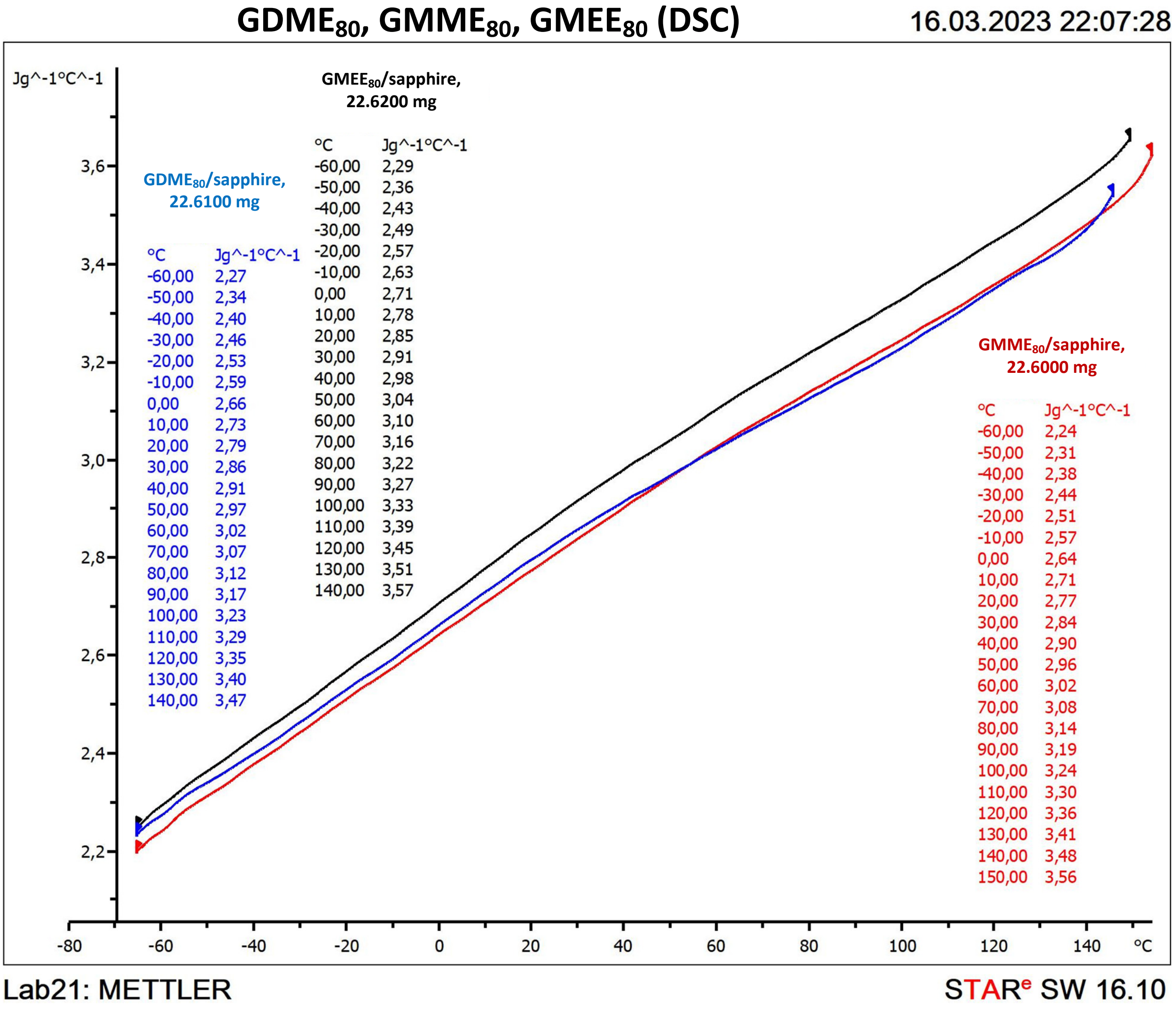

Figure S10. The DSC curves for $GDME_{80}$, $GMME_{80}$ and $GMEE_{80}$ samples (specific heat capacity measurement).

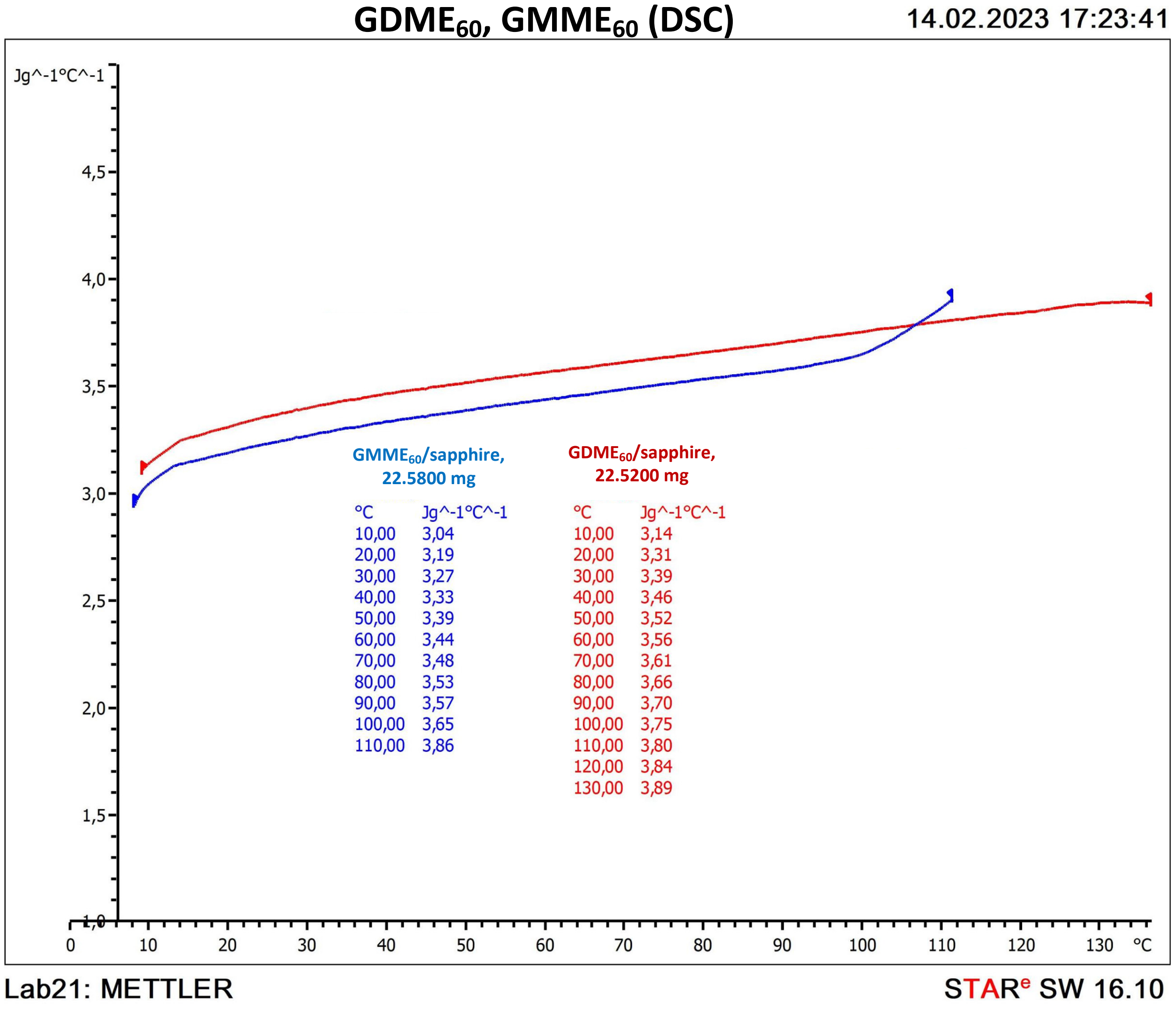

Figure S11. The DSC curves for $GDME_{60}$ and $GMME_{60}$ samples (specific heat capacity measurement).

Density measurements: calibration, raw data and uncertainties.

Density measurements

Apparatus: 'VIP-2MR' vibration densimeter ('Termeks', Tomsk, Russia).

Calibration:

a) dry air, T=20°C, individual values 0.0012; 0.0012; 0.0012 g mL^{-1}; reference value 0.0012 g mL-1.
b) distilled water (standard reference sample), T=20°C, individual values 0.9982; 0.9982; 0.9982 g mL^{-1}; reference value 0.9982 g mL^{-1}.

The raw data for the density of the glycerol ether aqueous solution samples is presented in the Table S1.

Table S1. The raw density data for the glycerol ether aqueous solution samples. MV – mean value, relSD – standard deviation expressed in %.

**GMEE, wt %**	**20**	**40**	**50**	**60**	**70**	**80**	**100**
density at 10oC, g cm-3	1,0373	1,0592	1,0657	1,0735	1,0773	1,0781	1,073
	1,0372	1,0593	1,0657	1,0736	1,0773	1,0782	1,073
	1,0372	1,0593	1,0658	1,0735	1,0772	1,0782	1,073
MV	1,0372	1,0593	1,0657	1,0735	1,0773	1,0782	1,0730
relSD	0,01%	0,01%	0,01%	0,01%	0,01%	0,01%	0,00%
density at 20oC, g cm-3	1,0272	1,0467	1,0544	1,0615	1,0651	1,0658	1,0602
	1,0272	1,0467	1,0543	1,0616	1,0652	1,0659	1,0603
	1,0271	1,0467	1,0543	1,0616	1,0652	1,0658	1,0603
MV	1,0272	1,0467	1,0543	1,0616	1,0652	1,0658	1,0603
relSD	0,01%	0,00%	0,01%	0,01%	0,01%	0,01%	0,01%
density at 40oC, g cm-3	1,0103	1,0268	1,0312	1,0377	1,0396	1,0406	1,0347
	1,0102	1,0269	1,0312	1,0377	1,0396	1,0406	1,0347
	1,0103	1,0269	1,0312	1,0377	1,0396	1,0406	1,0347
MV	1,0103	1,0269	1,0312	1,0377	1,0396	1,0406	1,0347
relSD	0,01%	0,01%	0,00%	0,00%	0,00%	0,00%	0,00%
**GMME, wt %**	**20**	**40**	**50**	**60**	**70**	**80**	**100**
density at 10oC, g cm-3	1,0335	1,069	-	1,0983	-	1,1192	1,1287
	1,0336	1,069	-	1,0983	-	1,1192	1,1287
	1,0336	1,069	-	1,0983	-	1,1192	1,1287
MV	1,0336	1,0690	-	1,0983	-	1,1192	1,1287
relSD	0,01%	0,00%	-	0,00%	-	0,00%	0,00%
density at 20oC, g cm-3	1,0261	1,06	1,075	1,0882	1,0991	1,1083	1,1165
	1,0262	1,0601	1,075	1,0883	1,0992	1,1083	1,1166
	1,0261	1,0601	1,075	1,0883	1,0992	1,1082	1,1166
MV	1,0261	1,0601	1,0750	1,0883	1,0992	1,1083	1,1166
relSD	0,01%	0,01%	0,00%	0,01%	0,01%	0,01%	0,01%
density at 40oC, g cm-3	1,008	1,039	-	1,064	-	1,0823	1,0913
	1,008	1,039	-	1,064	-	1,0823	1,0913
	1,008	1,039	-	1,064	-	1,0823	1,0913
MV	1,0080	1,0390	-	1,0640	-	1,0823	1,0913
relSD	0,00%	0,00%	-	0,00%	-	0,00%	0,00%
**GDEE, wt %**	**20**	**40**	**50**	**60**	**70**	**80**	**100**
density at 10oC, g cm-3	1,0162	1,0217	1,0132	0,9938	0,9635	1,0162	1,0217
	1,0162	1,0217	1,0132	0,9938	0,9636	1,0162	1,0217
	1,0162	1,0217	1,0132	0,9938	0,9636	1,0162	1,0217
MV	1,0162	1,0217	1,0132	0,9938	0,9636	1,0162	1,0217
relSD	0,00%	0,00%	0,00%	0,00%	0,01%	0,00%	0,00%
density at 20oC, g cm-3	1,0075	1,0099	1,0004	0,9815	0,9503	1,0075	1,0099
	1,0074	1,01	1,0005	0,9816	0,9503	1,0074	1,01

	1,0074	1,01	1,0005	0,9816	0,9503	1,0074	1,01
MV	1,0074	1,0100	1,0005	0,9816	0,9503	1,0074	1,0100
relSD	0,01%	0,01%	0,01%	0,01%	0,00%	0,01%	0,01%
density at 40oC, g cm-3	0,9889	0,9857	0,9737	0,9545	0,9226	0,9889	0,9857
	0,9889	0,9857	0,9737	0,9545	0,9226	0,9889	0,9857
	0,9889	0,9857	0,9737	0,9545	0,9226	0,9889	0,9857
MV	0,9889	0,9857	0,9737	0,9545	0,9226	0,9889	0,9857
relSD	0,00%	0,00%	0,00%	0,00%	0,00%	0,00%	0,00%
**GDME, wt %**	**20**	**40**	**50**	**60**	**70**	**80**	**100**
density at 10oC, g cm-3	1,0235	1,043	-	1,0543	-	1,0523	1,036
	1,0235	1,043	-	1,0543	-	1,0522	1,0359
	1,0235	1,0431	-	1,0543	-	1,0522	1,0359
MV	1,0235	1,0430	-	1,0543	-	1,0522	1,0359
relSD	0,00%	0,01%	-	0,00%	-	0,01%	0,01%
density at 20oC, g cm-3	1,0161	1,0329	1,0394	1,0426	1,0423	1,0393	1,0218
	1,0161	1,0329	1,0394	1,0426	1,0424	1,0393	1,0218
	1,0161	1,033	1,0393	1,0426	1,0424	1,0393	1,0218
MV	1,0161	1,0329	1,0394	1,0426	1,0424	1,0393	1,0218
relSD	0,00%	0,01%	0,01%	0,00%	0,01%	0,00%	0,00%
density at 40oC, g cm-3	0,9978	1,0104	-	1,0172	-	1,0133	0,9971
	0,9978	1,0105	-	1,0173	-	1,0132	0,9971
	0,9978	1,0105	-	1,0173	-	1,0132	0,9971
MV	0,9978	1,0105	-	1,0173	-	1,0132	0,9971
relSD	0,00%	0,01%	-	0,01%	-	0,01%	0,00%

Viscosity measurements: calibration, raw data and uncertainties.

Kinematic viscosity measurements

Apparatus: ‘VPZh-4’ glass capillary viscometer (‘Ekros’, Moscow, Russia) with a metrological certificate, KRIO-VIS-T-06-01 bath thermostate (‘Termeks’, Tomsk, Russia).

Calibration: for an additional control of the accuracy of the measurements, viscosities of lab-prepared distilled water were measured.

Measured values: 1.004 mm^2 s^{-1} at 20°C, 0.658 mm^2 s^{-1} at 40°C, 0.365 mm^2 s^{-1} at 80°C.

Reference values: 1.0034 mm^2 s^{-1} at 20°C, 0.6579 mm^2 s^{-1} at 40°C, 0.3643 mm^2 s^{-1} at 80°C.

The reference values were taken from Anton Paar open source (https://wiki.anton-paar.com/be-en/water/).

The raw data for the viscosity of the glycerol ether aqueous solution samples is presented in the Table S2.

Table S2. The viscosity raw data for the glycerol ether aqueous solution samples.

GMME, wt %	10	20	40	60	80	100
**Measurement temperature 20ºC**						
measured values, $mm^2\ s^{-1}$	-	1,84	3,64	8,18	19,78	50,50
	-	1,84	3,64	8,17	19,79	50,60
	-	1,85	3,67	8,17	19,87	50,56
mean value	-	1,84	3,65	8,17	19,81	50,55
standard deviation (absolute)	-	0,006	0,017	0,006	0,049	0,050
standard deviation (relative to mean value)	-	0,58%	0,47%	0,07%	0,25%	0,10%
**Measurement temperature 40ºC**						
measured values, $mm^2\ s^{-1}$	17,66	1,12	1,99	3,81	8,00	470,72
	17,42	1,12	2,00	3,84	8,00	471,63
	17,38	1,12	1,98	3,83	8,00	471,25
mean value	17,49	1,12	1,99	3,83	8,00	471,20
standard deviation (absolute)	0,151	0,000	0,010	0,015	0,000	0,457
standard deviation (relative to mean value)	0,87%	0,00%	0,50%	0,40%	0,00%	0,10%
**Measurement temperature 80ºC**						
measured values, $mm^2\ s^{-1}$	4,11	0,56	0,86	1,4	2,38	332,94
	4,11	0,56	0,86	1,4	2,38	332,72
	4,13	0,56	0,86	1,4	2,38	333,84
mean value	4,12	0,56	0,86	1,40	2,38	333,17
standard deviation (absolute)	0,01	0,00	0,00	0,00	0,00	0,59
standard deviation (relative to mean value)	0,00	0,00	0,00	0,00	0,00	0,00
**Measurement temperature -20ºC**						
measured values, $mm^2\ s^{-1}$	-	-	-	98,21	365,84	533,25
	-	-	-	98,23	365,07	533,28
	-	-	-	98,22	-	-
mean value	-	-	-	98,22	365,46	533,27
standard deviation (absolute)	-	-	-	0,010	0,544	0,021
standard deviation (relative to mean value)	-	-	-	0,01%	0,15%	0,00%

GMEE, wt %	20	40	60	80	100
**Measurement temperature 20ºC**					
measured values, $mm^2\ s^{-1}$	393,28	361,41	313,78	21,38	49,00
	393,91	361,63	316,28	21,31	49,00

	-	-	312,88	21,36	49,00
mean value	393,60	361,52	314,31	21,35	49,00
standard deviation (absolute)	0,445	0,156	1,762	0,036	0,000
standard deviation (relative to mean value)	0,11%	0,04%	0,56%	0,17%	0,00%
**Measurement temperature 40ºC**					
measured values, mm^2 s^{-1}	370,56	434,46	349,00	8,5	17,15
	370,57	435,31	349,03	8,5	17,15
	369,97	433,25	349,50	8,49	17,13
mean value	370,37	434,34	349,18	8,50	17,14
standard deviation (absolute)	0,344	1,035	0,280	0,006	0,012
standard deviation (relative to mean value)	0,09%	0,24%	0,08%	0,07%	0,07%
**Measurement temperature 80ºC**					
measured values, mm^2 s^{-1}	160,28	290,94	290,68	2,49	4,04
	160,63	290,82	290,59	2,49	4,04
	-	289,94	-	2,5	4,04
mean value	160,46	290,57	290,64	2,49	4,04
standard deviation (absolute)	0,247	0,546	0,064	0,006	0,000
standard deviation (relative to mean value)	0,15%	0,19%	0,02%	0,23%	0,00%
**Measurement temperature -20ºC**					
measured values, mm^2 s^{-1}	-	-	429,66	402,19	501,78
	-	-	429,25	399,69	502,28
	-	-	-	403,56	-
mean value	-	-	429,46	401,81	502,03
standard deviation (absolute)	-	-	0,290	1,962	0,354
standard deviation (relative to mean value)	-	-	0,07%	0,49%	0,07%

**GDME, wt %**	**20**	**40**	**60**	**80**	**100**
**Measurement temperature 20ºC**					
measured values, mm^2 s^{-1}	1,92	3,43	5,69	4,98	4,15
	1,91	3,48	5,65	4,99	4,15
	1,91	3,45	5,65	4,96	4,15
mean value	1,91	3,45	5,66	4,98	4,15

standard deviation (absolute)	0,006	0,025	0,023	0,015	0,000
standard deviation (relative to mean value)	0,30%	0,73%	0,41%	0,31%	0,00%
**Measurement temperature 40ºC**					
measured values, mm^2 s^{-1}	1,12	1,86	2,74	3,13	2,41
	1,12	1,86	2,79	3,15	2,41
	1,12	1,86	2,78	3,13	2,42
mean value	1,12	1,86	2,77	3,14	2,41
standard deviation (absolute)	0,000	0,000	0,026	0,012	0,006
standard deviation (relative to mean value)	0,00%	0,00%	0,96%	0,37%	0,24%
**Measurement temperature 80ºC**					
measured values, mm^2 s^{-1}	0,55	0,8	1,1	1,28	1,09
	0,55	0,8	1,09	1,25	1,08
	0,55	0,8	1,08	1,26	1,09
mean value	0,55	0,80	1,09	1,26	1,09
standard deviation (absolute)	0,000	0,000	0,010	0,015	0,006
standard deviation (relative to mean value)	0,00%	0,00%	0,92%	1,21%	0,53%
**Measurement temperature -20ºC**					
measured values, mm^2 s^{-1}	-	-	55,28	59,94	26,41
	-	-	55,25	59,99	26,44
	-	-	54,96	60,06	26,4
mean value	-	-	55,16	60,00	26,42
standard deviation (absolute)	-	-	0,177	0,060	0,021
standard deviation (relative to mean value)	-	-	0,32%	0,10%	0,08%

**GDEE, wt %**	**20**	**40**	**60**	**80**	**100**
**Measurement temperature 20ºC**					
measured values, mm^2 s^{-1}	719,81	749,25	6,52	7,02	4,16
	722,19	746,37	6,53	7	4,16
	725,66	-	6,53	7,01	4,15
mean value	722,55	747,81	6,53	7,01	4,16
standard deviation (absolute)	2,942	2,036	0,006	0,010	0,006

standard deviation (relative to mean value)	0,41%	0,27%	0,09%	0,14%	0,14%
**Measurement temperature 40ºC**					
measured values, $mm^2\ s^{-1}$	222,63	430,56	3,17	3,44	2,34
	222,32	430,81	3,17	3,45	2,34
	-	-	3,17	3,44	2,34
mean value	222,48	430,69	3,17	3,44	2,34
standard deviation (absolute)	0,219	0,177	0,000	0,006	0,000
standard deviation (relative to mean value)	0,10%	0,04%	0,00%	0,17%	0,00%
**Measurement temperature 80ºC**					
measured values, $mm^2\ s^{-1}$	168,88	290,19	1,23	1,32	1,07
	168,69	289,88	1,23	1,33	1,07
	-	292,34	1,23	1,33	1,07
mean value	168,79	290,80	1,23	1,33	1,07
standard deviation (absolute)	0,134	1,340	0,000	0,006	0,000
standard deviation (relative to mean value)	0,08%	0,46%	0,00%	0,44%	0,00%
**Measurement temperature -20ºC**					
measured values, $mm^2\ s^{-1}$	-	-	81,63	72,67	28,91
	-	-	81,62	72,7	28,91
	-	-	81,57	72,73	28,89
mean value	-	-	81,61	72,70	28,90
standard deviation (absolute)	-	-	0,032	0,030	0,012
standard deviation (relative to mean value)	-	-	0,04%	0,04%	0,04%
